# Supplementary material for: Assessment of knowledge, attitude and practice on first aid management of choking and associated factors among kindergarten teachers in Addis Ababa governmental schools, Addis Ababa, Ethiopia. A cross-sectional institution-based study
Source: PLoS One. 2021 Jul 30;16(7):e0255331. doi: 10.1371/journal.pone.0255331 (PMC8323944; doi:10.1371/journal.pone.0255331)
Supplement: S1 File — (DOCX) [file pone.0255331.s001.docx]

Annex I**:** Information sheet

**Addis Ababa University**

**College of Health Science**

**Department of Emergency medicine**

Good Morning/Good Afternoon!

This Study is prepared to Collect Data on assessment of knowledge, attitude and practice towards choking first aid among Government kindergarten teachers in Addis Ababa City.

My Name Is _____________________________________, I am conducting the research on the assessment of Knowledge, attitude and practice of choking first aid among kindergarten teachers in Bole,Gulele and Kirkos Sub Cities, Addis Ababa, Ethiopia.

The study is being conducted by Ali Maalim Issack, Addis Ababa University, Department of Emergency Medicine, in Postgraduate Program. The objective of this study is to assess knowledge, attitude and practice towards choking first aid among kindergarten teachers in governmental schools, Addis Ababa. The assessment is made for the partial fulfilment of Master’s Degree in Emergency Medicine and critical care nursing. The results of the study will be used as base line information to design appropriate intervention strategies to increase kindergarten teachers’ knowledge, attitude and practice of choking first aid. The questionnaire contains closed ended questions and will be provided in self-administered form. You are therefore kindly requested to provide genuine answers to the questions. The information you provide is confidential and is used only for the purpose of this study. If you have any question, don‘t hesitate to ask the data collector. Your cooperation and participation until the completion of the questionnaire is very necessary for the successful completion of the study.

Annex II: Consent form

In signing this document, I am giving my consent to participate in the study entitled “Assessment of knowledge attitude and practice towards choking first aid among Governmental kindergarten school teacher in Addis Ababa city”.

I have been informed that the purpose of this study is to assess knowledge, attitude and practice towards choking first aid among government kindergarten school teachers in Addis Ababa.

I have understood that participation in this study is entirely voluntarily. I have been told that my answers to the questions will not be given to anyone else and no reports of this study ever identify me in any way. I have also been informed that my participation or non-participation or my refusal to answer questions will have no effect on me.

I understood that participation in this study does not involve risks. I understood that Ali Maalim is the contact person if I have questions about the study or about my rights as a study participant.

We therefore ask your genuine willingness. However, you have the right to refuse if you are not voluntary to participate by making thick mark in -No’ in the box below.

Do you agree to participate Yes sign--------- No

If yes proceed to the next page

Thank you in advance for your cooperation

Data collectors Name____________________, date ____________sign: __________

Questionnaire code: _________________

Annex III: Questionnaire

**Part 1**

**Socio-demographiccharacteristics of kindergarten teachers in Addis Ababa**

| **Ser. no** | **Socio-Demographic** | **Response** | **Remark** |
| --- | --- | --- | --- |
| **1** | Sex | 1. Male 2. Female |  |
| **2** | Age | _______in years |  |
| **3** | Level of education | 1. Certificate 2. Diploma 3. Degree 4. Masters 5. Other (specify) ____ |  |
| **4** | Marital status | 1. Married 2. Single 3. Divorced 4. Widowed/ |  |
| 5 | Service year | 1. <1 yrs. 2. 1 - 5 yrs. 3. >5-10 yrs. 4. >10 |  |
| 6 | Previous first aid training | 1. Yes 2. No |  |

**Part II**

**Questions to assess kindergarten teacher’s choking first aid knowledge in Addis Ababa**

1 i) have you heard about choking first aid?

1. Yes B) No

ii) If yes for from which source you heard?

1. Media B) family members C) colleagues D) health professional E) others, specify

2, i) Do you know choking first aid?

1. Yes B) No

ii) If yes where you learn from?

1. From my previous first aid training B) from a friend C) from my studies D) from health professional E, others, specify________________

3, which of the following(s) is (are) the universal sign(s) of choking?

1. Coughing and/or crying B) knocking at the chest C) clinching at the throat D) wheezing E) A and D

4, which of the following student’s behaviors may led to choking? (Multiple answer is possible)

1. Playing football B) running while eating C) putting objects in their mouth D) playing, talking and laughing while eating

5, which of the following factor(s) led to choking among preschool children?

A, improper chewing of foods B) immature molars C) running with food in their mouth with subsequent incoordination between their swallowing reflexes and glottis closure D) adventurous nature E) all of the above

6, the following(s) are potential choking hazards except?

A) Hot dogs B) coins C) applesauce D) whole grapes E) popcorn

7, Golden time for providing choking first aid is____?

1. 2 minute B) 3 minute C) 4 minute D) 5 minute E) 1 hour

8, which of the following is a symptom(s) of complete airway obstruction?

A, crying loudly B) coughing c) inability to produce sound and cough D) blood in saliva

9, how can you prevent children from choking? (You can choose more than one answer)

1. Keep solid things away from children B) stop talking while eating C) I don’t know how to prevent child from choking D) proper chewing of food

10, symptoms of partial obstruction of airway?

1. Inability to produce sound B) wheezing C) unable to breath D) sneezing

11, is choking induced by aspiration of fluids?

1. Yes B) No C) Not sure

**Part III**

**Questions to assess attitude of kindergarten teachers towards choking first aid in Addis Ababa governmental KG schools.**

**Answer the following question by saying: -** strongly agree, agree, Disagree, strongly disagree

1, choking should need immediate management?

A, Agree B, Disagree C, strongly agree D, strongly disagree E, not sure

2, everybody should know about first aid management of choking?

A, strongly agree B, agree C, Disagree D, strongly disagree E, not sure

3, choking does not cause death or life threatening condition even if not treated

A, agree B, Disagree C, strongly agree D, strongly disagree E, not sure

4, it is possible to manage choking at the school without taking a victim to the health institution.

A, strongly agree B, Agree C, Disagree D, strongly disagree E, not sure

5, we should sweep our fingers blindly into throat of choked victim and take him/her to the health institution as soon as possible

A, strongly agree B, Agree C, strongly disagree D, Disagree E, not sure

6, you must not provide choking first aid without knowledge

A, Agree B, Disagree C, strongly agree D, strongly disagree E, not sure

7, if choking first aid is not given within golden time, it may led to death.

A, strongly agree B, Agree C, Disagree D, strongly disagree E, not sure

**Part IV**

**Questions to assess practice of kindergarten teachers on choking first aid in Addis Ababa**

**Practice**

1, i) had choking occurred in your presence outside school?

1. Yes B) No

ii) if yes have you provided choking first aid management?

1. Yes B) No

2, i) had choking occurred in front of you in the school compound?

1. Yes B) No

ii) if yes have you provided choking first aid immediately?

A) Yes B) No

iii) if No for Q 1 ii and Q 2 ii what is the reason for not providing choking first aid?

1. I don’t know choking first aid B) fear of law C) fear of complication D) fear of communicable disease transmission E) others specify____________

3, what do you do initially if you see a 4 year old child suddenly choking with food during lunch at your kindergarten school and he develop difficulty of breathing and speaking with complete obstruction of airway and food is not visible?(after calling ambulance)

A, giving him a glass of water B, do finger sweep to identify and remove object C, hitting at the back of neck D, abdominal thrust E, slapping at the back F, others, specify______

4, what will you do next if your initial procedure in Q 1 failed?

A, finger sweep B, sent to health institution C, chest thrust, D, Abdominal thrust E, others specify___

5, how many time(s) will you do the procedures you answered in Q 1 and 2 respectively?

A, 3 and 5 B, 2 and 10C, 3 and3 D, 5 and 5 E, 5 and 3

6, at which location of the body will you perform the procedure you answered in Q 1 and 2 respectively?

A, between breast bone and the base of the ribs B, below the shoulder and at the umbilicus C, between shoulder blades and the base of the ribs D, just below the neck and the base of ribs E, I don’t know

7, what do you do if you face a 5 years old child who suddenly develop choking during meal time in your kindergarten and develop difficulty of breathing and unable to talk with complete obstruction of airway, foreign body is visible and accessible?

A, talking him to health institution B, notifying school director and calling the parents

C, remove the foreign body by yourself D, hitting at the back of neck

E, giving a sip of water f, Abdominal thrust g, others, specify______

8, what do you do if child is choking and coughing?

A, chest thrust B, slapping at the back C, giving a glass of water and call ambulance

D, Abdominal thrust E, encourage him to cough and call ambulance

9, a child is choking in your kindergarten during a break time, one student came running and inform you, you run and see a child has difficulty of breathing and speaking, As you are proving choking first aid, his condition worsen and suddenly he lost consciousness and breathless. What will you do?

A, slapping at the back B, give two rescue breathing and do CPR C, do cardiopulmonary resuscitation D, contact responsible school authority E, I don’t know what to do F, others, specify________

10, a 9 months old boy of your neighbor is choking in your presence, he cannot cough, talk or breathe, the first thing you should do is_____________?

A, give abdominal thrust B, give back slaps C, put your finger in his mouth and do finger sweep to find out what is choking him D, call ambulance and wait for them to arrive

11, you are having a lunch with your friend who is also a KG teacher and he begun to choke on some food. He can’t speak, breath or cough up. What will you do to help your friend?

A, give him a glass of water B, give back slaps C, do abdominal thrusts D, put your finger down his throat to try to dislodge the obstruction of airway E, chest thrust

12, what will you do if a 15 years old girl suffered from choking while eating, she can be able to speak?

A, encourage her to cough B, help her to buckle her throat C, give her a water to drink D, give her a peace of injira

AMHARC VERSION OF Questionnaires

የፍቃደኝነት ስምምነት

**አዲስ አበባ ዩኒቨርስቲ**

**የጤና ሣይንስ ኮሌጅ**

**የኢመርጀንሲ ሜዲሲን ዲፖርትመንት**

እንደምን አደሩ/እንደምን ዋሉ , ስሜ ይባላል፡፡ በእውቀት አመለካከት እና በክህሎት ላይ ያተኮረ የመታነቅ የመጀመሪያ ደረጃ ህክምና እርዳታ አሰጣጥ በሚል ርዕስ ላይ በአዲስ አበባ ከተማ አስተዳደር በቦሌ ፣ በጉለሌ እና በቂርቆስ ክፍለ ከተማ በሚገኙ የመዋዕለ ህፃናት ትምህርት ቤቶች ውስጥ በሚሰሩ የተመረጡ የመዋለ ሕጻናት አስተማሪዎች ላይ በሚደረግ ጥናት መረጃ እየሰበሰብሁ እገኛለሁ፡፡

ጥናቱ እየተካሄደ የሚገኘው በአሊ ማሊም ይስሃቅ በአ.አ ዩኒቨርስቲ በድንገተኛ ትምህርት ክፍል የድንገተኛ ና ፅኑ ህሙማን ነርስ የሁለተኛ ዲግሪ ተመራቂ ተማሪ ነው፡፡ የዚህ ጥናት አላማ የአስተማሪወችን የመታነቅ የመጀመሪያ ደረጃ ህክምና እርዳታ አሰጣጥ በእውቀት፣ አመለካከትና ክህሎት ላይ ያላቸውን ክፍተት ጥናት ለማድረግና አስፈላጊውን የማስተካከያ ስርዓት ለሚመለከተው አካል ለማሳወቅ ነው፡፡ የጥናቱ ውጤት ተገቢ የሆነ ስትራቴጂ ዲዛይን ለማድረግ እና የመዋለ ሕጻናት አስተማሪዎችን እውቀት፣ አመለካከትና ክህሎት የመታነቅ የመጀመሪያ ደረጃ እርዳታ ላይ እውቀታቸውን ለማሳደግ እንደ መሠረታዊ መረጃ ያገለግላል፡፡ ቃለ መጠይቁ ዝግ እና ክፍት የሆኑ ጥያቄዎችን የሚያካትት እና በእርስዎ የሚሞላ ሆኖ የቀረበ ነው፡፡ ስለዚህ ለቃለ መጠይቁ ተገቢ የሆነ መልስ እንዲሰጡ በአክብሮት አጠይቆታለሁ፡፡

የሚሰጡት መረጃ በሚስጢር የሚያዝ እና ለዚህ ጥናት ብቻ በጥቅም ላይ የሚውል ይሆናል፡፡ ማንኛውም አይነት ጥያቄ ካሎት መረጃ ሰብሳቢውን ይጠይቁ፡፡ ለዚህ ጥናት ስኬታማነት እስከ ቃለ መጠይቁ መጠናቀቅ ድረስ ትብብሮት እና ተሳትፎዎት ወሳኝ ነው፡፡ ስለዚህ በጐ ፍቃደኝነቶን እጠይቃለሁ፡፡

ሆኖም ግን በዚህ ቃለ መጠይቅ ላይ ለመሳተፍ ካልፈለጉ በሚከተለው ሣጥን ውስጥ አይደለሁም የሚለውን ምልክት በማድረግ መተው ይችላሉ፡፡

ፍቃደኛ ነወት አዎ 
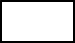
 አይደለም
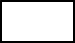


ለትብብርዎ በቅድሚያ እናመሰግናለን፡፡

የመረጃ ሰብሳቢው ስም ____________________ ቀን _________ ፊርማ__________________

የቃለ መጠይቅ ኮድ ___________________

**ቃለ መጠይቅ**

**ክፍል 1**

በአ.አ የሶሺዮ ዲሞግራፊክ ባህሪ ያላቸው የመዋለ ሕጻናት አስተማሪዎች

| ተ.ቁ. | ሶሺዮ ዲሞግራፊክ | ምላሽ | መግለጫ |
| --- | --- | --- | --- |
| 1 | ጾታ | 1. ወንድ 2. ሴት |  |
| 2 | እድሜ | ----- ዓመት |  |
| 3 | የትምህርት ደረጃ | 1. ሰርተፍኬት 2. ዲፕሎም 3. ዲግሪ 4. ማስተር 5. ሌሎች(ይግለጹ) |  |
| 4 | የጋብቻ ሁኔታ | 1. ያገባ 2. ያላገባ 3. የፈታ 4. መበለት |  |
| 5 | የአገልግሎት ዓመት | 1. ከ1 ዓመት በላይ 2. ከ1-5 ዓመት 3. ከ5-10 ዓመት 4. ከ10 ዓመት በላይ |  |

**ክፍል 2**

በአ.አ የመዋለ ሕጻናት አስተማሪዎችን የመታነቅ የመጀመሪያ ደረጃ እርዳታ አሰጣጥ ለማጥናት የቀረበ ጥያቄ

I. ከዚህ በፊት የመጀመሪያ ደረጃ እርዳታ ሥልጠና ወስደዋል ?

ሀ. አዎ ለ. አይደለም

II. መልሶት አዎ ከሆነ የመታነቅ የመጀመሪያ ደረጃ እርዳታ ያካተተ ነበር ?

ሀ. አዎ ለ. አይደለም

2.I ስለ መታነቅ የመጀመሪያ ደረጃ እርዳታ ሰምተው ያውቃሉ ?

ሀ. አዎ ለ. አይደለም

2.II መልሶት አዎ ከሆነ ከየትኛው ምንጭ ነው የሰሙት ?

ሀ. ሚዲያ ለ. ከቤተሰብ አባል ሐ. ኮሌጅ መ. የጤና ባለሙያ ሠ. ሌሎች ይግለጹ-----------------------

3.I የመታነቅ የመጀመሪያ ደረጃ እርዳታ ያውቃሉ ?

ሀ. አዎ ለ. አይደለም

III. መልሶት አዎ ከሆነ ከየት ነው የተማሩት ?

ሀ. ባለፈው ከወሰድኩት የመጀመሪያ ደረጃ እርዳታ ለ. ከጓደኞች ሐ. ከጥናቴ

መ. ከጤና ባለሙያ ሠ. ሌሎች ይግለጹ

4. ከሚከተሉት ውስጥ ዓለምአቀፋዊ የመታነቅ ምልክት የሆነው የትኛው ነው ?

ሀ. ማሳል እና/ወይም ማልቀስ ለ. ደረት ላይ መምታት ሐ. በጉሮሮ ላይ እጅን መለጠፍ መ. ማቃሰት ሠ. ሀ እና መ

5. ከሚከተሉት ውስጥ የትኛው የተማሪዎች ባህሪ ለመታነቅ ይዳርጋል? (ብዙ መልስ መስጠት ይቻላል፡፡)

ሀ. ኳስ መጫወት ለ. እየበሉ መሮጥ ሐ. ነገሮችን በአፍ ውስጥ መክተት መ. መጫወት ማውራት እና እየበሉ መሳቅ

6. ከሚከተሉት ምክንያቶች መካከል የትኛው የመዋለ ሕጻናት ተማሪ እንዲታነቅ ሊያደርግ ይችላል ?

ሀ. ምግብን ተገቢ ባልሆነ መንገድ ማኘክ ለ. ያልጠነከረ ጥርስ ሐ. ምግብ በአፋቸው ውስጥ አስቀምጠው መሮጥ መ. እዩኝ እዩኝ የማለት ባህሪ ሠ. ሁሉም

7. ከሚከተሉት ውስጥ ከአንዱ በስተቀር በባህሪያቸው መታነቅን የሚያስከትለው የትኛው ነው ?

ሀ. ሆት ዶግስ ለ. ሣንቲም ሐ. አፒልሶስ ሐ. ዎልግራፕስ መ. ፈንድሻ

8. የመታነቅ የመጀመሪያ ደረጃ እርዳታ ለመስጠት ወርቃማ ጊዜ የትኛው ነው ?

ሀ. 2 ደቂቃ ለ. 3 ደቂቃ ሐ. 4 ደቂቃ መ. 5 ደቂቃ ሠ. 1፡00 ሰዓት

9. ከሚከተሉት ውስጥ የትኛው ሙሉ ለሙሉ የመታነቅ ምልክት ነው ?

ሀ. በከፍተኛ ድምጽ ማልቀስ ለ. ማሳል ሐ. ድምጽ ማውጣት ማቃት መ. በምራቅ ውስጥ ደም መኖር

10. ልጆች እንዳይታነቁ እንዴት ልናደርግ እንችላለን (ከአንድ በላይ መልስ መምረጥ ይችላሉ)

ሀ. ጠጣር ነገሮችን ከልጆች በማራቅ ለ. ምግብ ሲበሉ እንዳያወሩ በማድረግ ሐ. ልጆች እንዳይታነቁ ምንም ማድረግ እንዳለብን እኔ አውቃለሁ መ. ምግብን በተገቢው ሁኔታ እንዲያገኙ በማድረግ

11. በከፊል የመታነቅ ምልክት የትኛው ነው ?

ሀ. ድምጽ ማውጣት አለመቻል ለ. ማቃሰት ሐ. መተንፈስ አለመቻል

መ. ማስነጠስ

12. ፈሳሽ ነገሮች መታነቅን ሊያስከትሉ ይችላሉ ?

ሀ. አዎ ለ. አይደለም ሐ. እርግጠኛ አይደለሁም

**ክፍል 3**

በአ.አ የኬጂ አስተማሪዎችን የመታነቅ የመጀመሪያ ደረጃ እርዳታ ለመገምገም የቀረበ ጥያቄ

የሚከተሉትን ጥያቄዎች በጣም እስማማለሁ እስማማለሁ አልስማማም በጣም አልስማማም በማለት ይመልሱ

1. መታነቅ ወዲያውኑ መረዳት የሚያሰፈልገው ክስተት ነው ?

ሀ. እስማማለሁ ለ. አልስማማም ሐ. በጣም እሰማማለሁ መ. በጣም አልሰማማም

2. ሁሉም ሰው ስለ መታነቅ የመጀመሪያ እርዳታ ህክምና ማውቅ አለበት ?

ሀ. በጣም እሰማማለሁ ለ. እስማማለሁ ሐ አልስማማም መ. በጣም አልሰማማም

3. መታነቅ እርዳታ ባይደረግም እንኳን ሞትን አያስከትልም

ሀ. እስማማለሁ ለ. አልስማማም ሐ. በጣም እሰማማለሁ መ. በጣም አልሰማማም

4. ተጐጂው ወደ ጤና ጣቢያ መውሰድ ሳያስፈልግ መታነቅን መርዳት ይቻላል

ሀ. በጣም እሰማማለሁ ለ. እስማማለሁ ሐ አልስማማም መ. በጣም አልሰማማም

5. ተጐጂው ወደ ጤና ጣቢያ ከመውሰዳችን በፊት ጣታችንን ወደ ጉሮሮው በመስደድ የታነቀውን ነገር ማስወገድ ይቻላል፡፡

ሀ. በጣም እሰማማለሁ ለ. እስማማለሁ ሐ አልስማማም መ. በጣም አልሰማማም

6. ያለ እውቀት የመታነቅ የመጀመሪያ ደረጃ እርዳታ መስጠት የለብዎትም

ሀ. እስማማለሁ ለ. አልስማማም ሐ. በጣም እሰማማለሁ መ. በጣም አልሰማማም

7. በወርቃማ ሰዓት ውስጥ በምግብ የመታነቅ የመጀመሪያ ደረጃ እርዳታ ካልተደረገ ለሞት ይዳርጋል

ሀ. በጣም እሰማማለሁ ለ. እስማማለሁ ሐ አልስማማም መ. በጣም አልሰማማም

ክፍል 4

በአ.አ የኬጂ አስተማሪዎችን የመታነቅ የመጀመሪያ ደረጃ እርዳታ ለመገምገም የቀረበ ጥያቄ

1. I. ባሉበት ቦታ ከትምህርት ቤት ውጪ የመታነቅ አደጋ አጋጥሞት ያውቃል ?

ሀ. አዎ ለ. አይደለም

II. መልሶት አዎ ከሆነ የመጀመሪያ እርዳታ አድርገዋል ?

ሀ. አዎ ለ. አይደለም

2. I. በትምህርት ቤት ቅጥር ግቢ ውስጥ በእርሶ ፊት መታነቅ ገጥሞት ያውቃል ?

ሀ. አዎ ለ. አይደለም

II. መልሶት አዎ ከሆነ ወዲያውኑ የመጀመሪያ እርዳታ አድርገዋል ?

ሀ. አዎ ለ. አይደለም

III. መልሶት ለጥያቄ ቁጥር 1 እና ቁጥር 2 አይ የሚል ከሆነ የመጀመሪያ ደረጃ እርዳታ ያልሰጡበት ምክንያት ምንድን ነው ?

ሀ. የመጀመሪያ ደረጃ የመታነቅ እርዳታ መስጠት አላውቅም

ለ. የሕግ ፍራቻ

ሐ. የውስብስብነት ፍራቻ

መ. የተላላፊ በሽታ ፍራቻ

ሠ. ሌላ ካለ ይግለጹ ------------------

3. በመዋለ ሕጻናት ውስጥ የ4 ዓመት ሕጻን በድንገት በምሳ ሰዓት በምግብ ታንቃ ቢያገኛት እና መተንፈስ እና መናገር ቢያቅታት ምን ያደርጋሉ? (ለአንቡላንስ ከደወሉ በኃላ)

ሀ. በብርጭቆ ውሃ መስጠት ለ. ምግቡን ለማውጣት ጣት መክተት

ሐ. ማጀራት መምታት መ. ሆድ መጫን ሠ. በጀርባ በኩል መምታት ሠ. ሌላ ካለ ይግለጹ

4. የጥያቄ ቁጥር 1 ሳይሳካ ቢቀር ምን ያደርጋሉ?

ሀ. ጣት መክተት ለ. ወደ ጤና ጣቢያ መላክ ሐ. ደረት መጫን መ. ሆድ መጫን

ሠ. ሌሎች ካሉ ይግለጹ

1. በጥያቄ ቁጥር 1 እና 2 ላይ የሰጡትን መልስ በተከታታይነት ምን ያህል ጊዜ ይሰጣሉ?

ሀ. 3 እና 5 ለ. 2 እና 10 ሐ. 3 እና 3 መ. 5 እና 5 ሠ. 5 እና 3

6. በተራ ቁጥር 1 እና 2 ላይ የሰጡት መልስ በቅደም ተከተልነት በየትኛው አካል ክፍል ላይ ፈጸማል;

ሀ. በጡት አጥንት እና በመገጣጠሚያ መካከል ለ. ከጉልበት በታች ሐ. በትከሻ እና በመገጣጠሚያ መካከል መ. ከአንገት በታች እና ከመገጣጠሚያ በታች ሠ. አላውቅም

7. በመዋለ ሕጻናት ውስጥ የ5 ዓመት ሕጻን ምግብ በምትበላበት ጊዜ በምግብ ታንቃ ብተገኝ እና መተንፈስ እና መናገር ቢያቅታት ምን ታደርጋህ ?

ሀ. ለጤና ተቋም መናገር ለ. ለት/ቤቱ ዳይሬተር መናገር እና ለቤተሰብ መደወል

ሐ. ጉዳዩን በራስ መፍታት መ. በስተኋላ በኩል አንገት መምታት ሠ. ትንሽ ውሃ መስጠት ረ. ሆድ መጫን ሰ. ሌላ ካለ ይግለጹ

8. ሕጻኑ የታነቀ እና የሚያስል ከሆነ ምን ያደርጋሉ ?

ሀ. ደረት መጫን ለ. ከኋላ በኩል መምታት መ. ትንሽ ውሃ መስጠት እና አንቡላንስ መጥራት ሠ. ሆድ መጫን ረ. እንዲያስለው ማበረታታት እና አንቡላንስ መጥራት

9. በእረፍት ሰዓት በመዋለ ሕጻናት ውስጥ አንድ ሕጻን በምግብ ቢታነቅ እና አንድ ተማሪ እየሮጠ መጥቶ ቢነግሮት እርስዎ ሮጠው ሕጻኑን መተንፍስ እና መናገር እንደማይችል አይተው የመጀመሪያ ደረጃ እርዳታ ሰተውት ሁኔታው ብሶበት ሕጻኑ ራሱን ቢስት እና መተንፈስ ቢያቅተው ምን ያደርጋሉ ?

ሀ. ከኋላ በኩል መምታት ለ. ሁለት ጊዜ እስትንፋስ መስጠት እና የደረት መጫን መስራት

ሐ. የደረት መጫንና ትንፋሽ መስጠትን መሥራት መ. ጉዳዩ የሚመለከተውን የት/ቤቱን አካል መጥራት ሠ. ምን መደረግ እንዳለበት አላውቅም ረ. ሌላ ካለ ይግለጹ

10. የ9 ወር የጐረቤቶት ሕጻን ልጅ እርስዎ ባሉበት ቦታ ቢታነቅ እና መሳል እና መናገር ወይም መተንፈስ ቢያቅታት መጀመሪያ የሚያደርጉት ነገር ምንድን ነው ?

ሀ. ሆድ መደገፍ ለ. ከጀርባ በኩል መምታት ሐ. ጣት በአፍ ውስጥ መክተት እና ምን እንዳለ መፈለግ መ. ለአንቡላን መደወል እና መጠበቅ

11. የመዋለ ሕጻናት አስተማሪ ከሆነ ጓደኛዎ ምሳ እየበሉ ቢታነቅ መናገር እና መተንፈስ ወይም ማሳል ቢያቅተው ጓደኝዎትን ለመርዳት ምን ያደርጋሉ ?

ሀ. በብርጭቆ ውሃ መስጠት ለ. በስተኃላ በኩል መምታት ሐ. ሆድ ላይ መደገፍ

መ. ጣቶችን ጉሮሮ ውስጥ መክተት ሠ. ደረት ላይ መደገፍ

12. የ15 ዓመት ሴት ልጅ ምግብ በምትበላበት ጊዜ ብትታነቅ ነገር ግን መናገር ብትችል ምን ያደርጋሉ?

ሀ. እንድታስል አደርጋታለሁ ለ. ጉሮሮዋን እንድትይዝ እረዳታለሁ ሐ. የሚጠጣ ውሀ እሰጣታለሁ መ. ትንሽ ምግብ እሰጣታለሁ
